# Nekrotisierende Skleritis nach Akanthamöbenkeratitis

**DOI:** 10.1007/s00347-020-01080-y

**Published:** 2020-03-11

**Authors:** Stefan J. Lang, Daniel Böhringer, Thomas Reinhard

**Affiliations:** Klinik für Augenheilkunde, Medizinische Fakultät, Universitätsklinikum Freiburg, Killianstr. 5, 79106 Freiburg, Deutschland

**Keywords:** Akanthamöben, Keratitis, Nekrotisierende Skleritis, Thermokauterisation, Amnionmembrantransplantation, Acanthamoeba, Keratitis, Necrotizing scleritis, Thermal cauterization, Amniotic membrane transplantation

## Abstract

Die noduläre Skleritis und die nekrotisierende Skleritis sind seltene Komplikationen der Akanthamöbenkeratitis. Wir präsentieren den Fall einer 61-jährigen Patientin, die seit mehr als 4 Monaten an einer persistierenden Keratitis am rechten Auge litt. Es wurde eine perforierende Limbokeratoplastik durchgeführt. Die Untersuchung des Hornhautexplantates zeigte Akanthamöbenzysten. In den folgenden 5 Monaten zeigte die Sklera rezidivierende Abszedierungen. Wir führten insgesamt 2 Thermokauterisationen und 3 Amnionmembrantransplantationen durch. Nach unserem Wissen ist dies der erste Fall einer Sklerokeratitis nach einer Akanthamöbenkeratitis, welcher mit einer Kombination aus Thermokauterisation und Amnionmembrantransplantation behandelt wurde. Weitere Studien sind notwendig, um dieses Verfahren als Alternative zur etablierten Kryotherapie zu untersuchen.

## Abstract (en)

Nodular scleritis and necrotizing scleritis are rare complications of acanthamoeba keratitis. This article presents the case of a 61-year-old female patient who had suffered from persistent keratitis in the right eye for more than 4 months. The patient was initially treated with propamidine isethionate and polyhexamethylene biguanide eye drops. A penetrating limbo-keratoplasty was performed. Examination of the corneal explant showed acanthamoeba cysts. In the following 5 months the sclera showed recurrent abscesses. A total of two thermal cauterizations and three amniotic membrane grafts were carried out. To our knowledge, this is the first case of sclerokeratitis after acanthamoeba keratitis which was treated with a combination of thermal cauterization and amniotic membrane transplantation. Further studies are necessary to investigate this procedure as an alternative to the established cryotherapy.

## Anamnese

Eine 61-jährige Patientin stellte sich aufgrund einer seit mehr als 4 Monaten anhaltenden Keratitis am rechten Auge in unserer Klinik vor. Die Patientin benutzte vor Beginn der Symptome Kontaktlinsen. Sie berichtete von einer kontinuierlichen Verschlechterung der Sehkraft und starken Schmerzen am rechten Auge. Die Patientin war zum Zeitpunkt der Vorstellung bereits mit Antibiotika‑, Propamidin-Isethionat- und Polyhexamethylenbiguanid-Augentropfen behandelt worden. Die Sehschärfe bei der Erstuntersuchung lag bei Fingerzählen. Die Untersuchung des vorderen Augenabschnittes zeigte eine Hornhauterosio und -trübung sowie Endothelbeschläge. Wir führten eine Limbokeratoplastik [4, 5] durch. Bei der histopathologischen Untersuchung des Hornhautschutzes wurden Akanthamöbenzysten festgestellt.

## Befund

**Figure 1:**
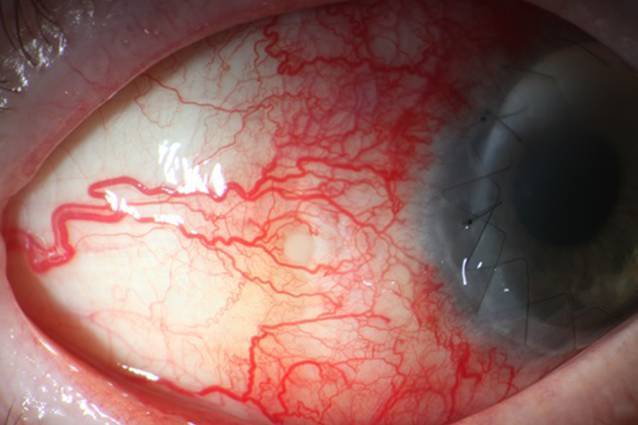

Zwei Wochen nach der Limbokeratoplastik wurde eine Abszedierung der Sklera bemerkt (Abb. 1).

## Diagnose

Nekrotisierende Skleritis nach Akanthamöbenkeratitis

## Therapie und Verlauf

**Figure 2:**
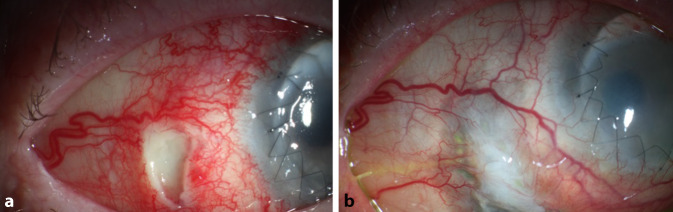

**Figure 3:**
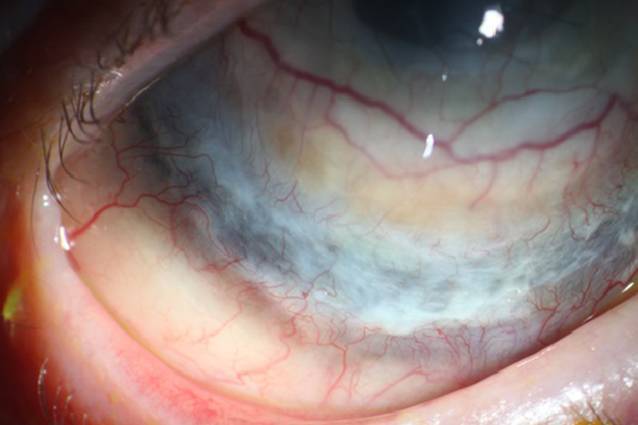

Es wurde eine Thermokauterisation des Abszesses durchgeführt (Abb. 2). Zudem erfolgte eine Amnionmembrantransplantation. Die topische Behandlung umfasste Propamidin-Isethionat, Polyhexamethylenbiguanid und Moxifloxacin-Augentropfen. Die systemische Behandlung wurde mit Methylprednisolon und Voriconazol durchgeführt. Im Laufe der folgenden 5 Monate kam es zu mehreren Episoden von skleralen Entzündungen und Einschmelzungen in verschiedenen Teilen der Sklera des rechten Auges. Es wurden mehrere Thermokauterisationen und Amnionmembrantransplantationen durchgeführt. Die Hornhauttransplantation zeigte während dieses Zeitraumes keine Anzeichen für ein Rezidiv der Akanthamöbenkeratitis. Letztendlich bestand eine zirkuläre Ausdünnung der Sklera um den Limbus (Abb. 3). Die histopathologische Untersuchung des Skleralgewebes ergab keinen Anhalt für Akanthamöben. In der weiteren Nachbeobachtung zeigte sich kein Rezidiv über die folgenden 12 Monate.

## Diskussion

Die nekrotisierende Skleritis ist eine seltene Komplikation der Akanthamöbenkeratitis [2]. In unserem Fall wurden bei der histopathologischen Untersuchung des Hornhautgewebes Akanthamöbenzysten nachgewiesen, aber nicht im Skleragewebe. Dies war auch der Fall in einem Bericht von Chatterjee et al. Die Autoren vermuten, dass nicht nur Akanthamöben, sondern auch eine immunologische Entzündung für das Einschmelzen des Gewebes verantwortlich sein könnte [1]. Daher sollte nicht nur eine Behandlung der Akanthamöben, sondern auch eine entzündungshemmende Behandlung durchgeführt werden. In unserem Fall zeigte das Hornhauttransplantat bei der Skleraverdünnung keine Anzeichen für ein Wiederauftreten der Akanthamöben. Der Einsatz von Amnionmembran bei postoperativer nekrotisierender Skleritis nach Mitomycin C-Behandlung wurde von anderen Arbeitsgruppen beschrieben [3]. Wir behandelten die Entzündung mit einem Wunddébridement und einer Thermokauterisation sowie mit zusätzlichen systemischen Steroiden. Zudem erfolgte eine Transplantation von Amnionmembran zur Oberflächenrekonstruktion. Dies musste mehrmals in Kombination mit einer kontinuierlichen Anti-Akanthamöben-Therapie (Voriconazol und topische Behandlung mit Propamidin-Isethionat, Polyhexamethylenbiguanid und Moxifloxacin-Augentropfen) wiederholt werden, um eine stabile Situation ohne Rezidiv der Skleritis zu erreichen. Nach unserem Wissensstand ist dies der erste Fall einer Akanthamöben-assoziierten Sklerokeratitis, welcher mit einer Kombination aus entzündungshemmender Therapie, Thermokauterisation und Amnionmembrantransplantation behandelt wurde. Diese Behandlung könnte in ausgewählten Fällen eine Alternative zur etablierten Kryotherapie sein und sollte in weiteren Studien evaluiert werden.

## Fazit für die Praxis

Die nekrotisierende Skleritis ist eine seltene Komplikation der Akanthamöbenkeratitis. Eine Behandlung mittels einer Kombination aus entzündungshemmender Therapie, Thermokauterisation und Amnionmembrantransplantation kann in schweren Fällen notwendig sein.
